# Diagnostic value of metagenomic next-generation sequencing in atypical brucellosis: a case report

**DOI:** 10.3389/fmed.2025.1652671

**Published:** 2025-10-10

**Authors:** Dungaowa Ao, Xinle Li, Guoliang Zhang, Huiyu Ma, Lijuan Yang, Hui Tian, Shuxia Ao, Jin Feng, Wanru Geng

**Affiliations:** ^1^Affiliated Hospital, Inner Mongolia Minzu University, Tongliao, China; ^2^The Fourth Affiliated Hospital, Anhui Medical University, Hefei, China; ^3^Tongliao Mongolian Medical Hospital, Tongliao, China

**Keywords:** brucellosis, submandibular space infection, metagenomic next-generation sequencing, *Brucella suis*, high-throughput sequencing

## Abstract

**Background:**

Brucellosis with atypical presentations, such as submandibular abscess without fever, is frequently misdiagnosed.

**Methods:**

Metagenomic next-generation sequencing (mNGS) was applied to pus samples from a 47-year-old female with a treatment-refractory submandibular abscess and a history of livestock exposure; results were confirmed serologically.

**Results:**

Within 48 h, mNGS identified *Brucella suis*—representing, to our knowledge, the first reported afebrile submandibular infection caused by this pathogen. Targeted therapy with doxycycline and rifamycin led to symptom resolution within 6 days.

**Conclusion:**

This case highlights that mNGS, combined with a thorough epidemiological history, can resolve diagnostic dilemmas in atypical brucellosis, guide precise treatment, and mitigate antibiotic misuse.

## Background

Brucellosis is a global zoonotic disease, with more than 500,000 cases reported worldwide each year (the actual incidence rate is even higher) (1). In China, more than 95% of the disease burden is in northern regions such as Inner Mongolia, and the peak incidence is closely related to the livestock breeding season (2, 3). The *Brucella* genus includes *Brucella melitensis* (65–70%), *Brucella abortus* (20–25%), *Brucella suis* (<5%). There are significant differences in their host adaptability and virulence factors (4). In China, brucellosis is mainly prevalent in sheep. Due to host specificity and geographical distribution limitations, Brucellosis infections in pigs are reported less frequently at present. The pathogen can be transmitted through skin and mucous membrane contact, ingestion of contaminated dairy products or inhalation of aerosols. The typical symptoms are wave fever, excessive sweating and joint pain. However, about 15–20% of cases present with atypical site infections (such as central nervous system infections and soft tissue abscesses) as the initial symptom (5).

## Introduction

Here we report a case of brucellosis misdiagnosed as submandibular space infection. The correct diagnosis was attributed to metagenomic next-generation sequencing (mNGS) technology, which provides detailed information on the microbial genome sequence (6). This unbiased high-throughput sequencing method can accurately diagnose by simultaneously and independently sequencing thousands to billions of DNA fragments to identify the total DNA or RNA content of all known pathogenic microorganisms (7).

## Case description

**Figure 1:**
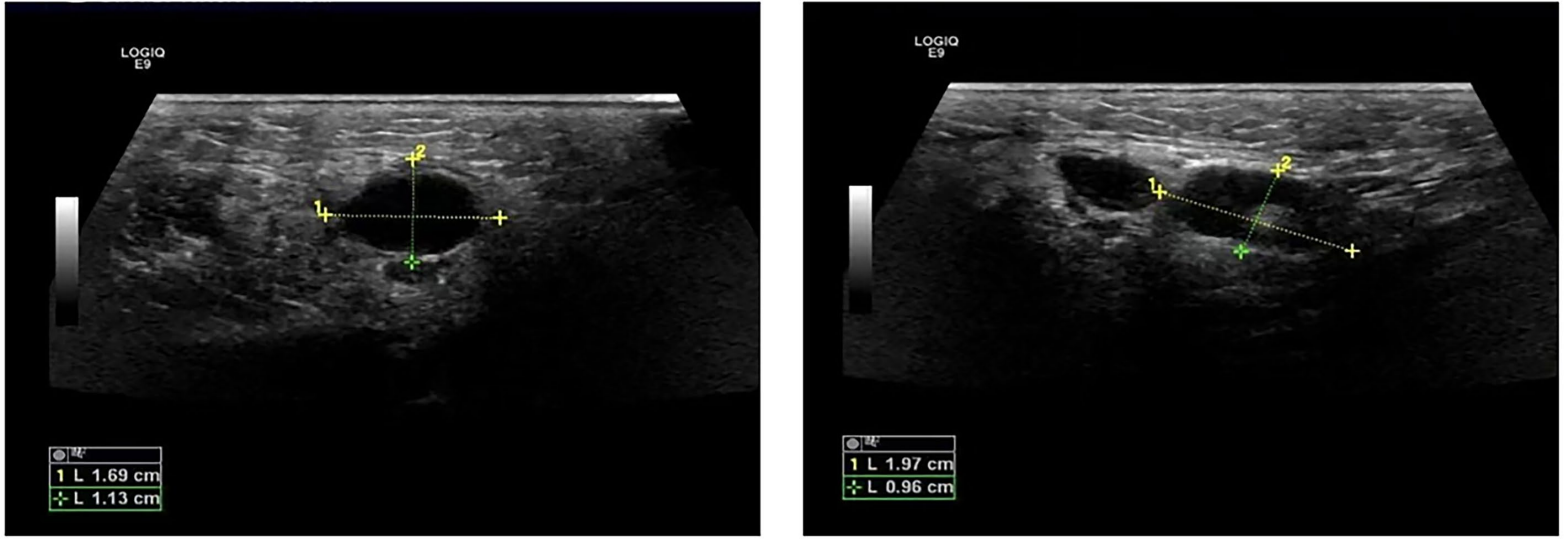
Ultrasound images of abscess.

**Figure 2:**
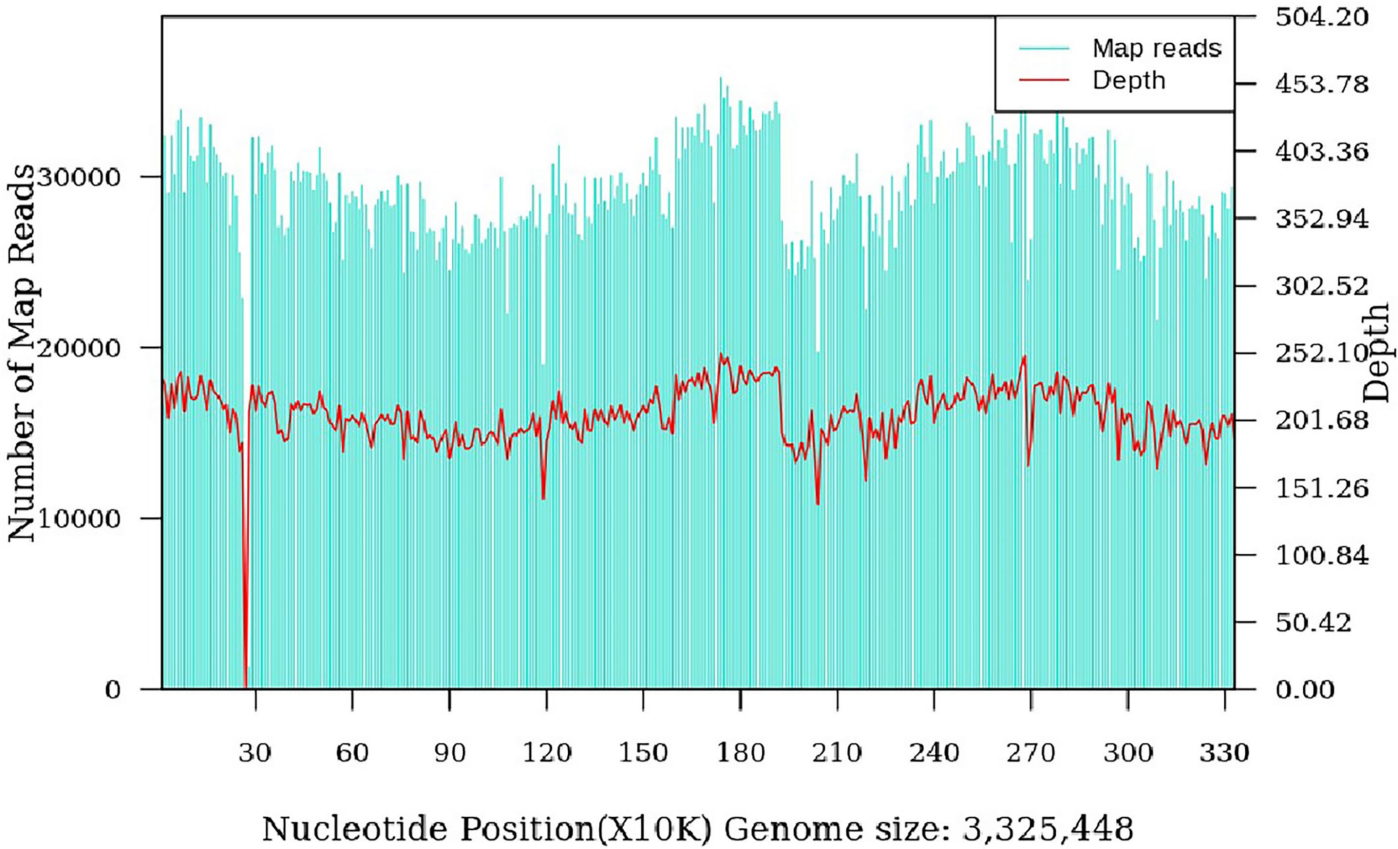
*Brucella suis* genome coverage map (coverage: 98.94%).

The patient was a 47-year-old female with a right maxillofacial abscess accompanied by distension and pain for 14 days. On November 22, 2024, the patient visited the local hospital’s Department of stomatology and was initially diagnosed with submandibular space infection. After local puncture and pus drainage, intravenous infusion of drugs such as clindamycin hydrochloride, levofloxacin and metronidazole for 3 days, the local symptoms worsened. On November 25, 2024, the patient visited the Oral and maxillofacial surgery outpatient Department of our hospital. The blood routine test did not show a positive result. The ultrasound examination indicated that a hypoechoic mass could be seen in the superficial part of the right masseter muscle of the patient, approximately 1.7*1.1 cm in size, with a clear boundary, regular shape, and uniform internal echo. No obvious blood flow signal was observed within it. Multiple lymph nod-like echoes could be seen in the right submandibular gland area, approximately 2.0*1.0 cm in size, with a clear structure and no obvious blood flow signal. Specialized physical examination indicated that the swelling range in the right submandibular area was approximately 2.0*1.5 cm (as shown in Figure 1), with local skin redness and swelling, elevated local skin temperature, tenderness (+), local fluctuation sensation, and slightly limited occlusal function. The re-diagnosis was submandibular space infection. Intravenous infusion of clindamycin, levofloxacin, ornidazole and other drugs for 3 days, but the therapeutic effect was not good. On November 29, 2024, the patient was referred to the Department of Infectious Diseases of our hospital. Based on her long-term epidemiological history of contact (including feeding and delivering) with livestock such as cattle and sheep, brucellosis is highly suspected. The suspension was sent to Jilin Jinyu Medical Laboratory for sequencing of the next generation of the metagenome (mNGS) the results showed that the relative abundance of *Brucella* with sequence number of 9133191 was 99.97%, and the coverage of *Brucella suis* with sequence number of 8525228 was 98.94%. Supplementary Brucella test tube agglutination method test suggests Brucella test tube agglutination 1:200++++. The blood culture and drug sensitivity tests were completed, indicating that the pathogenic bacterium was *Brucella*. Combined with the epidemiological history, metagenomic next-generation sequencing (mNGS) (as shown in Figure 2) and other auxiliary examination results, the diagnosis was modified to brucellosis. Surgical dressing changes were performed daily, and intravenous infusion of doxycycline 0.1 g q12h and rifamycin 0.5 g q12h was carried out. After 6 days of anti-brucellosis treatment, the patient’s symptoms were relieved and the patient was discharged. Blood cultures were negative at the first, third and sixth month after discharge (see Table 1).

**Table 1:** Laboratory test.

Test item	Day1	Day5	Day8	Day13
WBC (3.5–9.5/L)	9.36*10^9^	8.41*10^9^	5.83*10^9^	3.88*10^9^
NEUT% (40.0–75.0%)	55.3	76.8	60.1	42.8
NEUT# (1.8–6.3/L)	5.17*10^9^	6.46*10^9^	3.50*10^9^	1.66*10^9^
LYMPH% (20.0–50.0%)	31.6	15.3	23.3	39.4
LYMPH# (1.1–3.2/L)	2.96*10^9^	1.29*10^9^	1.36*10^9^	1.53*10^9^
MONO% (3.0–10.0%)	10.9	6.9	14.6	13.7
MONO# (0.10–0.60/L)	1.02*10^9^	0.58*10^9^	0.85*10^9^	0.53*10^9^
HGB (115–150 g/L)	124	123	108	105
HCT (0.35–0.45 L/L)	0.401	0.390	0.346	0.337
MCHC (316-354 g/L)	309	315	312	312
RDW-CV (12.0–14.3%)	15.3	15.6	15.5	14.9
RDW-SD (37.1–49.2 fL)	50.5	51.1	50.4	48.6
IG% (0.16–0.61%)	1.2	0.6	0.3	0.5
IG# (0.01–0.03/L)	0.11*10^9^	0.05*10^9^	0.02*10^9^	0.02*10^9^
K (3.5–5.3 mmol/L)	–	3.94	3.94	4.64
Hs-CRP (0–10.0 mg/L)	–	4.65	8.15	5.34

## Discussion

This case reports a case of brucellosis with infection in a rare site as the initial symptom and without fever, presenting as a submandibular space abscess without typical fever symptoms. Although brucellosis is relatively common in epidemic areas, its clinical manifestations are atypical brucellosis and diagnostic delays caused by traditional culture methods, highlighting the diagnostic challenges of zoonotic brucellosis. However, metagenomic next-generation sequencing (mNGS) can identify pathogens as early as possible and prompt the treatment plan to be rapidly adjusted from broad-spectrum antibiotics to targeted therapy with doxycycline combined with rifamycin, which aligns with the standard recommended regimen outlined in the World Health Organization’s guidelines for the treatment of human brucellosis and is in line with the strategic goal of the World Health Organization to curb antibiotic resistance (8, 9).

The pathogenic mechanism of *Brucella suis* in this case is consistent with the results of molecular studies in recent years. Two studies in 2024 revealed that the T4SS secretion system of *Brucella suis* promotes soft tissue colonization by regulating host cell apoptosis, providing a molecular mechanism explanation for the formation of local abscesses (10, 11). Furthermore, the patient’s long-term occupational exposure to cattle and sheep—including feeding and assisting with deliveries—supports the hypothesis of pathogen invasion through skin breaches or aerosols, with subsequent macrophage-mediated lymphatic spread to the submandibular space, particularly in the context of potential predisposing factors, such as the patient’s known hypertension, which may be associated with altered immune function.

However, the traditional culture methods have a misdiagnosis rate as high as 25–40% (12, 13) due to the slow growth of *Brucella* (requiring 5–7 days) and interspecific phenotypic overlap. This limitation is consistent with the conclusion of a 2022 study, which confirmed that mNGS can significantly reduce medical costs through rapid detection (specificity >99%) and early optimized treatment (6, 14).

This study is the first to report brucellosis with the main clinical manifestation of submandibular space infection, which expands the clinical pedigree of brucellosis involving rare anatomic sites and fills the literature gap of brucellosis involving rare sites of head and neck. This case suggests that even in non-epidemic areas, there should be diagnostic vigilance for unexplained soft tissue infections (especially those with a history of contact with livestock), and brucellosis should also be included in the differential diagnosis (15); this finding underscores the pivotal role of technological innovation in clinical practice. By enabling rapid pathogen identification and guiding optimal treatment, mNGS plays a crucial role in addressing the global challenge of antimicrobial resistance. However, the need to further explore the cost-effectiveness of their application in low- and middle-income countries has led to mNGS reasons for not being widely used (6, 16); Moreover, this case exemplifies the necessity of a multidisciplinary approach for complex infection management, highlighting the urgent need to establish standardized guidelines that enhance collaboration and improve patient outcomes. Finally, a standardized multidisciplinary diagnosis and treatment framework needs to be constructed, and clinical education on atypical brucellosis manifestations should be strengthened to optimize diagnosis, treatment and follow-up, and ultimately reduce the global burden caused by misdiagnosis of zoonoses and the abuse of antibiotics (8).

## Limitations

This study has several limitations. First, the findings are based on a single case report, which limits the generalizability of our conclusions. Second, while mNGS proved decisive in this instance, its higher cost and limited accessibility in low-resource settings may restrict its widespread adoption in areas where brucellosis is endemic. Future multi-center studies with larger sample sizes are needed to validate our findings and further evaluate the cost-effectiveness of mNGS in the management of atypical brucellosis.

## Patient perspective evaluation

Following an accurate diagnosis and targeted treatment, the patient’s symptoms resolved rapidly, thereby avoiding a prolonged period of misdiagnosis and ineffective therapy. This experience, as a livestock farmer, heightened her awareness of occupational exposure risks, prompting a commitment to implement stricter protective measures and undergo regular physical examinations in the future.

## Data Availability

The datasets presented in this study can be found in online repositories. The names of the repository/repositories and accession number(s) can be found in the article/supplementary material.
